# Prevalence of avian paramyxovirus type 1 in Mallards during autumn migration in the western Baltic Sea region

**DOI:** 10.1186/1743-422X-10-285

**Published:** 2013-09-12

**Authors:** Conny Tolf, Michelle Wille, Ann-Katrin Haidar, Alexis Avril, Siamak Zohari, Jonas Waldenström

**Affiliations:** 1 Centre for Ecology and Evolution in Microbial model Systems (EEMiS), Linnæus University, Kalmar SE-391 82, Sweden; 2 Department of Virology, Immunology and Parasitology, National Veterinary Institute (SVA), Ulls väg 2B, Uppsala 751 89, Sweden

## Abstract

**Background:**

Newcastle disease virus (NDV) is the causative agent of the Newcastle disease, a severe disease in birds associated with substantial economic losses to the poultry industry worldwide. Sweden is situated along the Western European waterfowl flyway and applies a non-vaccination policy combined with directives of immediate euthanisation of NDV infected flocks. During the last decades there have been several outbreaks with NDV in poultry in Sweden. However, less is known about the virus prevalence in the wild bird population including waterfowl, a well-established reservoir of avian paramyxovirus type 1 (APMV-1), the paramyxovirus serotype that include pathogenic NDV.

**Methods:**

The survey constituted of 2332 samples from Mallards (*Anas platyrhynchos*), trapped in the southern part of Sweden during autumn migration in 2010. These samples were screened for APMV-1 by real-time reverse transcription PCR, and viral strains from positive samples were isolated and characterized by sequence analysis of the fusion gene and by phylogenetic analysis.

**Conclusions:**

Twenty of these samples were positive for APMV-1, hence a virus prevalence of 0.9% (95% Confidence Interval [95% CI]=0.54%, 1.35%). The highest APMV-1 prevalence was detected in juvenile Mallards sampled in November (n=887, prevalence 1.24% ([95% CI])=0.67%, 2.24%). Sequence analysis and evaluation of phylogenetic relatedness indicated that isolated APMV-1 strains were lentogenic, and phylogenetically most closely related to genotype Ib strains within the clade of class II viruses. The sampling system employed enabled us to follow APMV-1 infections and the shedding of one particular viral strain in one individual bird over several days. Furthermore, combining previous screening results with the APMV-1 detections in this study showed that more than 50% of Mallards that tested positive for APMV-1 RNA were co-infected with influenza A virus.

## Background

Newcastle disease is a highly contagious, infectious disease in birds with recurrent outbreaks in poultry all over the world. The infection is caused by the Newcastle disease virus (NDV), more formally termed avian paramyxovirus type I (APMV-1), which is a member of the *Avulavirus* genus in the *Paramyxoviridae* family. This virus family is composed of ten different serotypes (APMV-1 to APMV-10), most of which are exclusive to birds [1]. Members of *Paramyxoviridae* have a single stranded, non-segmented, negative-sense RNA genome containing six open reading frames (ORFs) that encode a nucleocapsid protein (NP), a phosphoprotein (P), a matrix protein (M), a fusion protein (F), a hemagglutinin-neuraminidase (HN), and the RNA-dependent RNA polymerase (L). HN and F proteins are involved in host cell attachment and membrane fusion, respectively, during infection [2].

The F protein is expressed as a precursor (F0) that needs to be activated by proteolytic processing into active F1 and F2 [3]. The primary structure of the F0 cleavage site is an important factor associated with pathogenicity, which in turn is closely related to viral tissue tropism [4]. NDV are further divided into three different pathotypes, with classification criteria partly based on amino acid sequence of the F0 cleavage site. Velogenic and mesogenic pathotypes have several basic amino acid residues at their F0 cleavage site, which enable furin-like proteases expressed in multiple tissue types of the host to provide the maturation cleavage that is essential for viral progeny to become infectious [5,6]. Lentogenic viruses, on the other hand, encode F0 precursors with a cleavage site that lacks basic residues, and consequently, these viruses are restricted to replication in tissues of the infected birds that express trypsin-like enzymes, including the respiratory and gastro-intestinal tracts [7]. If introduced into chicken flocks, velogenic and mesogenic strains can cause severe outbreaks with high mortality rates, sometimes as high as 100%. These highly virulent strains are considered such a risk for the agricultural economy that NDV is included in the OIE-list of diseases that are considered ‘specific hazards’ by the World Organization for Animal Health.

There is substantial genetic variation within the APMV-1 serotype, and viral strains are subdivided into two major clades termed class I and class II [8,9]. One feature that distinguishes these clades is genome size, where class I viruses have a slightly larger genome (15 198 nucleotides) compared to class II strains (15 186 or 15 192 nucleotides). Previously, class I and II viruses have, based on sequence variation of the F gene, been subdivided into nine and eleven genotypes, respectively [8]. However, according to a recent proposal of a unified nomenclature for APMV-1 based on the F gene sequence, the class I clade consist of one single genotype while class II is composed of fifteen genetic groups [10]. Other features that differ between the classes of APMV-1 are pathogenicity and host range. Class I viruses are almost exclusively of the lentogenic type and detected in wild waterfowl, whereas a majority of velogenic strains, often isolated during outbreaks in poultry, belong to class II [11]. The reservoir hosts and transmission routes of viruses that become highly virulent in chicken are not well characterized, although reports from Australia seem to indicate that lentogenic strains circulating in wild birds may transform into velogenic variants when introduced into poultry [12,13]. In addition, the transition from a lentogenic to a velogenic pathotype in chickens has been demonstrated experimentally [14].

Wild waterfowl is also the main reservoir for influenza A virus (IAV). Naturally, there is a significant chance of birds being co- or superinfected with both AMPV-1 and IAV. The dynamics between these two viruses has been investigated experimentally in embryonated chicken eggs [15], but much less is known about corresponding dynamics in the wild bird population. Up until now, the prevalence of APMV-1 in wild birds has often been discovered as a consequence of failed attempts to determine the subtype of isolated IAV strains [16], rather than actively screening birds for the virus. We have conducted long-term surveillance for IAV in waterfowl, particularly Mallards (*Anas platyrhynchos*), in Sweden from 2002 to present [17,18]. Similar to other study systems, putative APMV-1 viruses have been isolated occasionally when propagating IAV-positive samples in embryonated chicken eggs, but the prevalence and patterns of occurrence of APMV-1 has not been investigated in detail. Prompted by the isolation of APMV-1 from IAV samples, a real-time reverse transcription PCR-based surveillance for APMV-1 in wild migratory waterfowl was set up to gain information regarding virus prevalence in these birds. The goals were to answer (A) how prevalent APMV-1 are in migratory Mallards on this migration route, (B) to investigate the pathotypes and phylogenetic relatedness of isolated APMV-1 viruses, and (C) assess the frequency of Mallards co-infected with APMV-1 and IAV.

## Material and methods

### Bird sampling

Wild Mallards were captured in a live-duck trap at the Ottenby Bird Observatory, on the island of Öland, Sweden during October and November of 2010. The trap was emptied once a day and captured wild birds as well as sentinel ducks, held in a secluded compartment of the trap to attract the wild birds, were sampled for avian viruses including APMV-1 and IAV. Samples from individual birds were taken either by swabbing the cloacae or by collecting fresh faeces at the bottom of the single-use boxes, according to a well-established system that has been described previously [19]. In addition to sampling, Mallards were also ringed, measured and weighed, and their sex and age determined. Immediately after sampling, the swabs were placed in transport media (Hanks balanced salt solution containing 0.5% lactalbuminm, 10% glycerol, 200 U/ml penicillin, 200 μg/ml streptomycin, 100 U/ml polymyxin B sulphate, and 250 μg/ml gentamycin, and 50 U/ml nystatin; Sigma) and stored at - 70°C until analysed.

### Extraction of viral RNA and rRT-PCR

RNA was extracted from 100 μl of samples using the MagNA Pure 96 isolation system and the MagNA Pure 96 DNA and Viral NA Large Volume Kit (Roche). For real-time reversed transcription PCR (rRT-PCR), 2 μl of each sample were screened for APMV-1 (amplifying 121 base pairs of the matrix gene) and influenza A virus (amplifying 101 base pairs of the matrix segment) by using the One-step RT-PCR kit (Qiagen), and established TaqMan-based real-time PCR protocols for each virus [20,21]. The allantoic fluid of eggs inoculated with APMV-1 and IAV, respectively, were used as positive controls.

### Virus isolation

In order to isolate virus, 100 μl of the positive swab samples, as determined by rRT-PCR, were inoculated in embryonated specific pathogen free (SPF) chicken eggs following established protocols [22]. Successful virus isolation was initially determined by hemagglutination tests, and later verified by nucleotide sequencing.

### Nucleotide sequencing

Viral RNA for sequencing was extracted from allantoic fluid of inoculated eggs using EZ1 BioRobot system and the EZ1 Virus Mini Kit v2.0 (Qiagen). Viral RNA was eluted in a final volume of 50 μl. A 1595 base pair long fragment (corresponding to nucleotide positions 4595 to 6191 in the genome of the LaSota strain, GenBank accession AF077761) of isolated APMV-1 strains were amplified with primers NDV+47Fw (5’-ATGGGCTCCAGACCTTCTACCAAGA-3’) and NDV+1671Rev (5’-TTGTAGTGGCTCTCATCTGATCTAGAGTAT-3’). Resulting amplicons were gel purified (Wizard SV Gel and PCR Clean-Up System, Promega) and cloned into pGEM-T cloning vector according to the manufacturer’s instruction (Promega). Single bacterial clones encoding the APMV-1 F gene fragment were detected by PCR by using primers corresponding to the T7 and SP6 promoter regions of the cloning vector. Amplicons from multiple clones encoding the F gene of the same virus isolate were purified (see above) and sequenced using the T7 and the SP6 primer. Sequences generated in this study were deposited in GenBank with accession numbers KC631386- KC631395.

### Phylogenetic analyses

The F gene nucleotide sequences were edited and assembled in Geneious version 6.0.6 [23], and aligned to sequences available in GenBank using the MAFFT method [24]. This software was also used for determining pairwise sequence identity of F gene sequences from virus isolates. Following removal of columns containing gaps from the alignment, a 1595 bp nucleotide fragment of the F gene was used in phylogenetic analysis. In the analysis, a subset of APMV-1 sequences was selected from GenBank based on their similarity, as indicated by BLAST, to viral isolate sequences of this study. F gene sequences representing distinct clades in a recently proposed APMV-1 genotype nomenclature were retrieved from the Diel et al. [10]. Phylogenetic relationships between viral sequences were inferred using the maximum likelihood method implemented in the SeaView software version 4.4.0 [25]. For the analysis, the Tamura-Nei substitution model including estimates of gamma-distributed rate heterogeneity among sites and proportion of invariable sites (i.e. TN93 G+I) was used [26]. Statistical support for nodes in generated tree was estimated by nonparametric bootstrapping using 100 pseudoreplicates [27]. The resulting phylogenetic tree was visualized using the MEGA software version 5.2 [28].

### Statistical analyses

Confidence intervals of a proportion, i.e. the virus prevalence, were estimated using the modified Wald method implemented in GraphPad Prism version 6.01 (GraphPad Software, San Diego, USA). In these analyses, the modified Wald method and a 95% confidence interval was used to quantify uncertainty [29]. Variation in prevalence between months (October and November) and age-classes (juvenile and adult) were evaluated statistically using random permutation tests, since the small sample size did not allow classical t-tests for comparison of proportions. The observed difference in proportion was compared to the 95% CI of the difference expected under the null hypothesis H0 (i.e. no difference between groups). The confidence intervals were established under 100,000 random permutations. The possibility of a relationship between APMV-1 and IAV infection in a given animal was examined using classical tests of independence in two-way tables. The Fisher's exact test [30] was used in these analyses because the expected numbers in each cell of the contingency table were small.

Since a given individual may appear several times in the cells of the contingency table, this could bias both the prevalence estimates (mean and CI) and the related test’s outputs. In particular, repetitions of a given individual for different co-infection may lead to overestimation of both the population prevalence and the correlation between APMV-1 and IAV infection. To be confident in the prevalence and test interpretation, 1000 random samples including only one observation per individual were generated. From these samples we computed the mean prevalence for each virus, the 95% CI of the means and the median of the p-values of the tests performed on each of these 1,000 samples.

## Results

### APMV-1 prevalence in Mallards during autumn migration

During October and November in 2010, 2332 samples from Mallards were collected and screened for APMV-1 by rRT-PCR (Table 1). Among these samples were twenty identified as APMV-1 positive, hence an overall prevalence of 0.9% (95% Confidence Interval [95% CI]=0.54%, 1.35%). When restricting the prevalence estimation to unique observations in each individual, the prevalence increases slightly (1.03%, 95% CI=[0.77%, 1.39%]). The prevalence did not differ between October and November (100,000 random permutations, observed difference of proportions = −0.0054, 95% CI of the expected distribution under H0 [−0.0076, 0.0075]). In addition, corresponding statistical analysis of prevalence in samples from birds of different age-classes showed that there was no significant difference in prevalence between juvenile and adult birds (100,000 randomization permutations, observed difference in proportion = 0.0064, 95% CI of the expected distribution under H0 [0.0058, 0.0088]).

**Table 1:** APMV-1 prevalence in migrating Mallards in the western Baltic Sea region in 2010

**Sampling**		**APMV-1 prevalence**	
**(time & age group)**	***n***	**%**	**95% CI**
October	1297	0.62	0.29-1.24
Juvenile	820	0.85	0.38-1.79
Adult	341	0.29	<0.01-1.81
Age undetermined	136	0.00	0.00-3.30
November	1035	1.16	0.64-2.04
Juvenile	887	1.24	0.67-2.24
Adult	92	0.00	0.00-4.81
Age undetermined	56	1.78	<0.01-10.34

The sampling system utilized in this study, which included trapping, sampling and subsequent release of birds, enabled multiple sampling of individual birds during their migratory stopover period. Among sampled birds, four individuals tested positive for APMV-1 on more than one occasion (Table 2). From two of these individuals (ring number 90A87768 and 90A87615), three APMV-1 positive samples were collected from each bird during a 4-day period, spanning late October and early November. However, of these positive samples only one isolate was obtained from each bird. From two other ducks (ring number 90A87537 and 90A87823) APMV-1 positive samples were collected two and four days apart, respectively. From the former bird only one isolate was obtained, while two isolates were generated from samples of the juvenile male 90A87823, hence enabling a genetic comparison of virus from October 31 to that from November 3 (Table 2, see further under the phylogenetic analysis paragraph).

**Table 2:** Repeated APMV-1 detection in individual Mallards

					NDV-PCR^*^
**Ring number**	**Age**	**Sex**	**Sample ID**	**Sample date**	**(Ct-value)**^**†**^
90A87768	Juvenile	Female	122419	2010-10-30	35.0
			124187	2010-10-31	38.5
			124282	2010-11-01	26.6
90A87615	Juvenile	Female	124228	2010-11-01	38.2
			124344	2010-11-02	36.0
			124418	2010-11-03	37.2
90A87823	Juvenile	Male	124127	2010-10-31	35.0
			124461	2010-11-03	35.6
90A87537	Juvenile	Female	124911	2010-11-08	39.0
			125006	2010-11-10	37.0

^*^NDV is used instead of APMV-1 in the name of the method to comply with the terminology used in the reference where this method was first reported [21].

^†^The cycle threshold value from the rRT-PCR screening.

### Characterization of APMV-1 isolates

Out of twenty rRT-PCR positive samples, ten were successfully inoculated in eggs (Table 3). Among these isolates nine were taken from juvenile birds, while the tenth sample was taken from a bird whose age was not determined. Near complete F gene sequences and BLAST analyses revealed that all isolates belong to the class II clade of APMV-1. Pairwise comparison of nucleotide sequence identity of these sequences showed that 124110, 124127 and 124461 had an identity ranging from 99.7 to 99.9%, whereas the identity to remaining sequences (124987, 124911, 124329, 124418, 124282, 124345 and 124265) ranged between 98.0 and 98.5%. Correspondingly, within this larger group of sequences the similarity ranged between 98.8 to 100%. Further sequence comparisons including analyses of the F0 protein cleavage site, according to deduced amino acid sequences, suggested that none of the APMV-1 isolates were of the velogenic pathotype (i.e., with F0 cleavage site including multiple basic amino acids and a phenylalanine at position 117). Among the Swedish isolates, the ^112^GKQGR*L^117^ motif were found in 7 out of 10 strains, whereas the ^112^EKQGR*L^117^ sequence was found in remaining isolates (Table 3).

**Table 3:** APMV-1 isolates obtained in this study

**Host**		**APMV-1 isolate**					
**Species**	**Age**	**Sample ID**	**Isolate name**	**Class**	**Genotype**	**Fusion cleavage site**	**GenBank accession**
Mallard	Juvenile	124110	APMV-1/mallard/Sweden/124110/2010	II	Ib	EKQGR*L	KC631386
Mallard	Juvenile	124127	APMV-1/mallard/Sweden/124127/2010	II	Ib	EKQGR*L	KC631387
Mallard	Undetermined	124265	APMV-1/mallard/Sweden/124265/2010	II	Ib	GKQGR*L	KC631388
Mallard	Juvenile	124282	APMV-1/mallard/Sweden/124282/2010	II	Ib	GKQGR*L	KC631389
Mallard	Juvenile	124329	APMV-1/mallard/Sweden/124329/2010	II	Ib	GKQGR*L	KC631390
Mallard	Juvenile	124345	APMV-1/mallard/Sweden/124345/2010	II	Ib	GKQGR*L	KC631391
Mallard	Juvenile	124418	APMV-1/mallard/Sweden/124418/2010	II	Ib	GKQGR*L	KC631392
Mallard	Juvenile	124461	APMV-1/mallard/Sweden/124461/2010	II	Ib	EKQGR*L	KC631393
Mallard	Juvenile	124911	APMV-1/mallard/Sweden/124911/2010	II	Ib	GKQGR*L	KC631394
Mallard	Juvenile	124987	APMV-1/mallard/Sweden/124987/2010	II	Ib	GKQGR*L	KC631395

### Phylogenetic analysis

**Figure 1:**
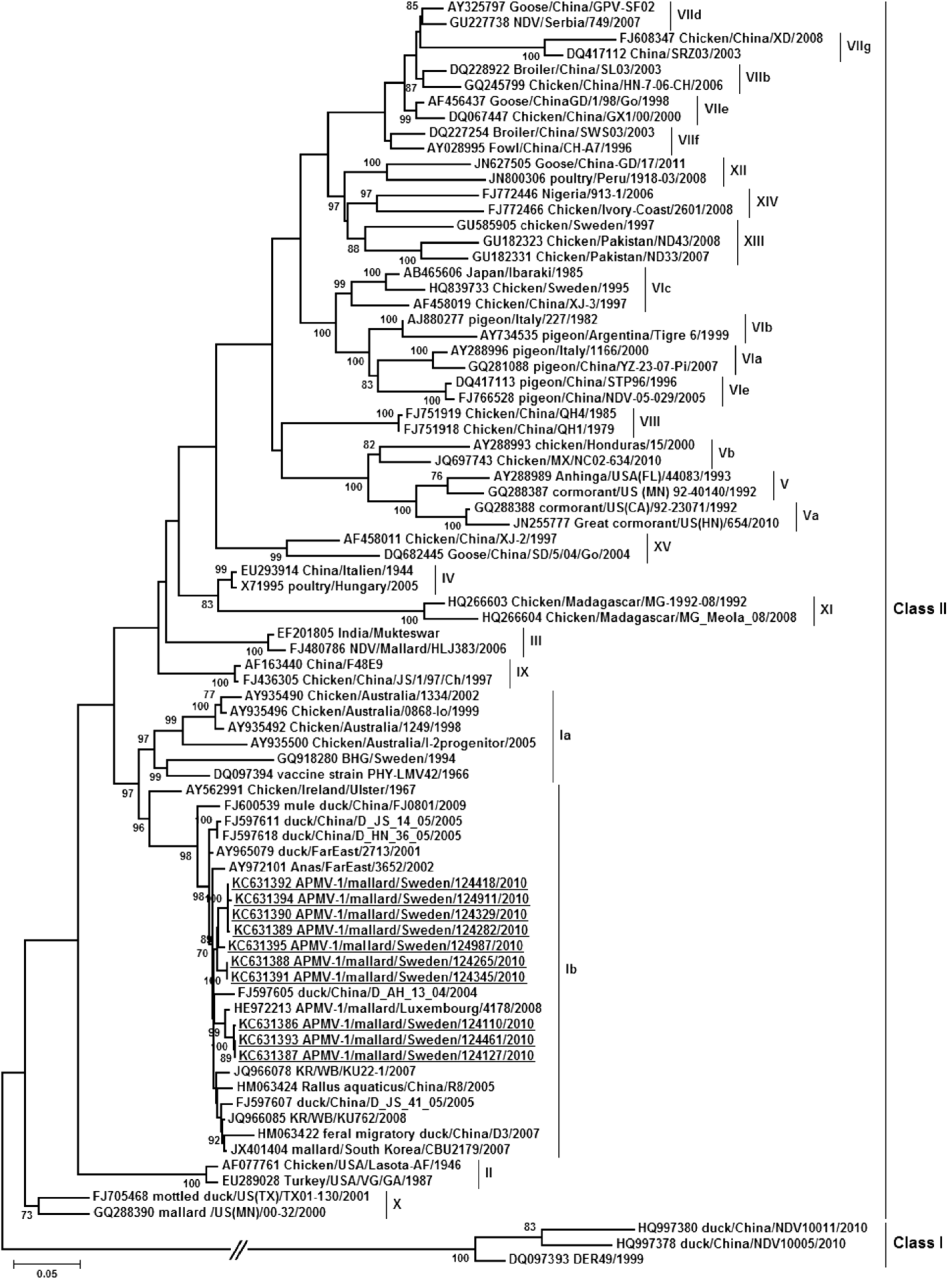
**Phylogenetic tree based on the partial nucleotide sequence of the Fusion gene of APMV-1.** Viral sequences generated in this study (underlined) and previously published sequences are denoted by their GenBank accession number followed by isolate names. The reference strains were adapted from a recently suggested nomenclature for APMV-1 [10]. Class I and class II viruses as well as genotype I to XV, including sublineages, within the class II clade are indicated by roman numerals. Statistical supports of ≥ 70% for inferred nodes are displayed. The branch between class I and class II clades of viruses has been truncated in order to limit the tree size.

Analysis of the phylogenetic relationships between the F gene sequences reported in this study, with those available in public databases, including Class I and Class II viruses, showed that the sequences from the Swedish isolates clustered with genotype Ib sequences within the class II clade (Figure 1). Corresponding to the distinction between sequences based on pairwise comparisons, within genotype Ib the Swedish isolates are divided into two clades with the majority of sequences forming a distinct genetic group, whereas the remaining three strains cluster with sequences from Mallard sampled in Luxemburg in 2008 and duck sampled in China in 2004.

### Mallards co-infected with APMV-1 and IAV

**Figure 2:**
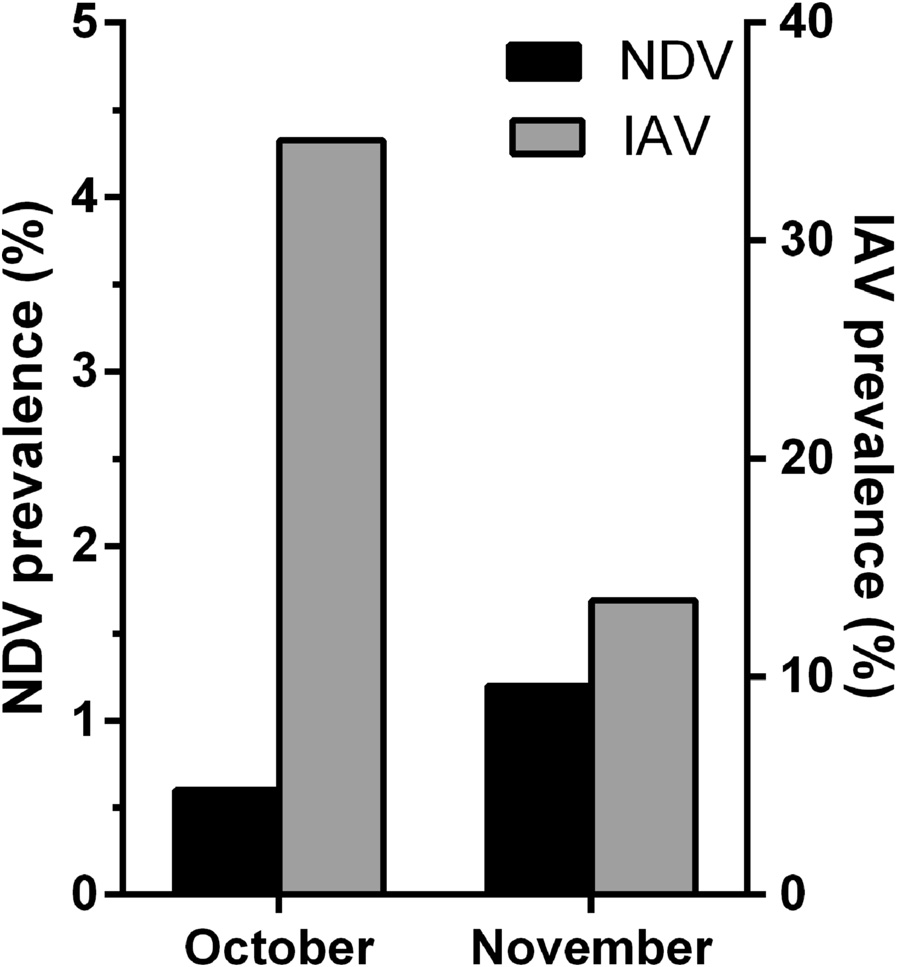
**Comparison of viral prevalence in Mallards.** Bars show the NDV and the IAV prevalence in Mallards during autumn migration in 2010.

Eleven of twenty samples positive for APMV-1 were also positive for IAV (Table 4). Nine of the co-infected birds were juveniles. Overall, the total prevalence of IAV in Mallards, regardless of age-classes, as determined by the rRT-PCR, was approximately 34% in October and 13% in November, respectively (Figure 2). Among the 2332 observations, 1734 samples were negative for both APMV-1 and IAV, while 9 and 578 samples were positive for APMV-1 and IAV, respectively. Accounting for repeated sampling of the same individual, these observations corresponds to a prevalence of 1.03% (95% CI=[0.77%, 1.39%] for APMV-1 and 23.5%, (95% CI=[22.0%, 25.1%]) for IAV. Further, 11 of these infected birds were co-infected with APMV-1 and IAV. Using the whole dataset, i.e. by including all observations in a given animal, we found a significant relationship between the APMV-1- and the IAV-infection status (Fisher exact test, p=7.10e-03). The absolute frequency of co-infections (n=11) was higher than the expected absolute frequency under the null hypothesis of independence between infections with these viruses (n=5.05). However, this result should be interpreted with caution as we found the median p-value of the tests performed over the 1000 samples, based on unique observation per individual, to be barely significant (p=0.054).

**Table 4:** Mallards co-infected with APMV-1 and IAV

**Sample-ID**	**Age**	**Sample date**	**Sex**	NDV-PCR^* ^**(Ct-value)**	**IAV-PCR (Ct-value)**
120930	Juvenile	2010-10-13	Male	31.8	24.2
120932	Adult	2010-10-13	Male	40.0	29.2
122310	Juvenile	2010-10-29	Male	29.8	35.0
122419	Juvenile	2010-10-30	Female	35.0	29.7
124110	Juvenile	2010-10-31	Female	37.3	25.2
124187	Juvenile	2010-10-31	Female	38.5	32.6
124228	Juvenile	2010-11-01	Female	38.2	37.1
124329	Juvenile	2010-11-01	Female	31.4	37.6
124418	Juvenile	2010-11-03	Female	37.2	38.0
124883	Juvenile	2010-11-08	Male	40.0	36.2
124987	Juvenile	2010-11-10	Male	29.0	38.0

^*^The PCR method is termed NDV to comply with the name used in the reference where this method was first established [21].

## Discussion

Previous surveillance studies indicate that waterfowl are an important reservoir of lentogenic class I and class II APMV-1 strains world-wide. All Scandinavian countries aside from Denmark employ a NDV non-vaccination policy, and rely instead on high biosecurity with poultry separated from wild birds, in addition to veterinary inspections and rapid diagnostics techniques for virus detection. Ongoing outbreaks are stamped out by euthanizing infected flocks, followed by disinfection of animal facilities. Despite the ban on vaccination, there have been few outbreaks in Scandinavia compared to other parts of the world, including Asia and Africa, where NDV are endemic in poultry. Two relatively recent NDV outbreaks in Sweden, one in 1995 and another one in 1997, were caused by velogenic class II strains of genotype VIc and genotype XIII, respectively ([31,32], see also in Figure 1). These particular genotypes have been circulating world-wide for decades, causing numerous outbreaks. Sweden is located in the north-western Baltic Sea region, and has a long coast-line along the Western European waterfowl flyway. It has previously been shown that Mallards from the eastern Baltic region migrate along the Swedish coast to their wintering grounds in Western Europe [33].

During the sampling period from October to November 2010, just over 2300 samples were collected and screened for presence of APMV-1. In total, less than one percent of the samples were APMV-1 positive, hence, a prevalence considerably lower than the total prevalence of 11.8% observed in waterfowl in Finland in 2010 [34]. In the same study it was shown that 4.8% of Mallards were infected. Possibly, observed differences in prevalence may in part be due to the fact that different rRT-PCR methods were used in our study [21] and the study by Lindh et al. [35]. It is also interesting to note, based on the results reported in the Finnish study, that the prevalence in Eurasian teal *Anas crecca* was higher than in Mallards [34]. It is not known whether this is due to differences in host susceptibility to the virus, or if it has more to do with differences in behaviour, including migration and breeding.

Comparisons of APMV-1 prevalence in Mallards of different age classes indicated that there is no significant difference in the probability of being infected. This observation does not agree with results obtained in a recent Australian study examining the relation between age and infection frequency [36]. In the Australian study, based on multivariate analysis of NDV infections in Plumed whistling ducks (*Dendrocygna eytoni*), for which the overall virus prevalence was 4.2%, the odds of being infected were approximately three times higher in juveniles compared to adult birds. It is possible that the limited dataset in our study, especially the number of APMV-1 infected adult birds, affects the outcome of the statistical analysis.

One advantage with the sampling system used at Ottenby, with daily sampling and subsequent release of sampled birds, is that it makes it possible to recapture individual birds and sample them several times during their stopover at the site. Among sampled birds in this study, four individuals that were positive on the first sampling occasion were later resampled within a two to four day period. Thus, in more extensive studies in the future, resampling systems may contribute to give a better understanding of the progression of infections in wild birds. Sequence analyses of the F gene sequence of isolates obtained from one of the ducks in this study seem to indicate that the same viral strain was shed during a four day period. To our knowledge this is the first observation of an APMV-1 infection in wild birds followed for several days.

Virus isolates were generated from ten out of twenty APMV-1 positive samples. As indicated by the deduced amino acid sequence of the F0 cleavage site, none of the APMV-1 isolates seem to be of the velogenic type. Corresponding results from screenings of wild birds have been reported previously, both in North America, Japan and Europe [16,37,38]. However, for an absolute confirmation of virus pathotype, tests in chicken embryos or chickens such as MDT (mean death time) and IVPI (intravenous pathogenicity index), respectively, needs to be performed [39].

The phylogenetic analysis of APMV-1 isolates showed that F gene sequences clustered with those of class II viruses. This is consistent with previous reports of a predominance of class II viruses in wild birds [8,16,34,36]. According to a recently suggested nomenclature of virus genotypes, class II viruses are divided into fifteen distinct clades [10]. Using this classification, the F0 sequences obtained in this study belong to genotype Ib, and cluster, within this clade, most closely with viral strains from wild waterfowl sampled in Luxembourg from 2006 to 2008 [40], and to viral sequences from birds sampled at live bird markets in China [41]. It is not presently known whether this vast geographic distribution of genetically related sequences is primarily a product of bird migration, or if lentogenic APMV-1 of particular genotypes are constantly circulating at different locations world-wide. However, the integration of several different migration routes of, for example Eurasian teal [42] carrying class II viruses of genotype I [34], including the north-west European flyway and the Mediterranean flyway, might, at least partly, contribute to the wide-spread distribution of certain virus genotypes.

It is worth pointing out that the detection of predominantly genotype Ib viruses is different from observations in recent North American studies detecting predominantly genotype II viruses in wild birds [8,16]. Possibly, differences dominating genotypes circulating in Northern Europe and North America is related to the geographical distance and the obstacle imposed by the Atlantic Sea, hence a phenomenon partly related to the well-established distinction between North American and Eurasian IAV sequences [43]. Among viral strains previously reported from Sweden, the sequences of this study is most closely related to a strain from a Black-Headed Gull (*Larus ridibundus*), found in the Ia clade, sampled at the Ottenby Bird Observatory in 1994 [44].

Superinfections and co-infections of the host with two or more pathogens may complicate studies of a certain pathogen’s effect on its host, but can also offer a valuable opportunity to explore particular properties unique to one of the pathogens during co-infection, and to evaluate the contribution of such factors to observed prevalence and virulence. Co-infections involving APMV-1 and IAV have been observed previously in North America and Europe [16,34]. In this study, more than half of the APMV-1 infected Mallard were simultaneously infected with IAV and statistical analyses suggest, although barely significant in this limited dataset, that the frequency of co-infection might be higher than expected. This is in contrast to previous observations with virus cross-species inhibition between NDV and IAV during co- and superinfection in experiments using embryonated chicken eggs [15,45]. Although these studies fail to provide a conclusive picture, the order and time of infection as well as the virulence of included virus strains seem to be significant for the outcome of experimental co-infections. A number of mechanisms have been put forward as possible explanations to observed virus induced viral cross-species inhibition, including attachment interference, differences in replication speed and intracellular interference in the form of virus-induced interferon production [46]. However, in wild birds infected with low pathogenic viruses, the cross-species relationship between viruses in a particular host might be different.

Another noticeable observation is the significant difference in APMV-1 and IAV prevalence in sampled ducks. APMV-1 and IAV have both negative-sense single-stranded RNA genomes with hemagglutinin and neuraminidase virion surface structures that attach to sialic acid moieties of host cell surface molecules. Also, for both viruses, waterfowl is a central reservoir for maintaining a constitutive circulation of virus. However, an important distinction between APMV-1 and IAV is their genome organization: The APMV-1 genome is consists of one continuous, linear RNA molecule, whereas IAV has a segmented genome consisting of eight linear RNA molecules. Possibly, the advantages with a segmented genome, including the capacity for genome reassortment, is a contributing factor to the higher IAV prevalence compared to that of APMV-1 in Mallards.

## Conclusions

In conclusion, approximately one percent of the Mallard population sampled during autumn migration at Öland, Sweden, was infected with class II APMV-1s. All virus isolates were of the lentogenic type and phylogenetically closely related to genotype I viruses from central and northern Europe, but clustered also with viruses isolated at live bird markets in the south eastern parts of China. Additional studies examining the circulation of APMV-1s in different wild avian species are essential in order to understand viral evolution and epidemiology.

## Competing interests

The authors declare that they have no competing interests.

## Authors’ contributions

CT and AKH designed the experiments and carried out the rRT-PCR screening. CT carried out sequence analysis and phylogenetic analysis. CT and MW drafted the manuscript. AA contributed with statistical analysis of prevalence data. SZ was involved in experimental design, and in revision of the manuscript. JW was involved in planning the study and in revision of the manuscript. All authors have read and approved the final manuscript.
